# Supplementary material for: Causal Relationship between Aging and Anorexia Nervosa: A White-Matter-Microstructure-Mediated Mendelian Randomization Analysis
Source: Biomedicines. 2024 Aug 16;12(8):1874. doi: 10.3390/biomedicines12081874 (PMC11351342; doi:10.3390/biomedicines12081874)
Supplement: Supplementary file 1 [file biomedicines-12-01874-s001.zip › Supplementary Figure/Figure S4/Singe SNP plot of MR analysis for TL to ubm-b-1687.pdf]

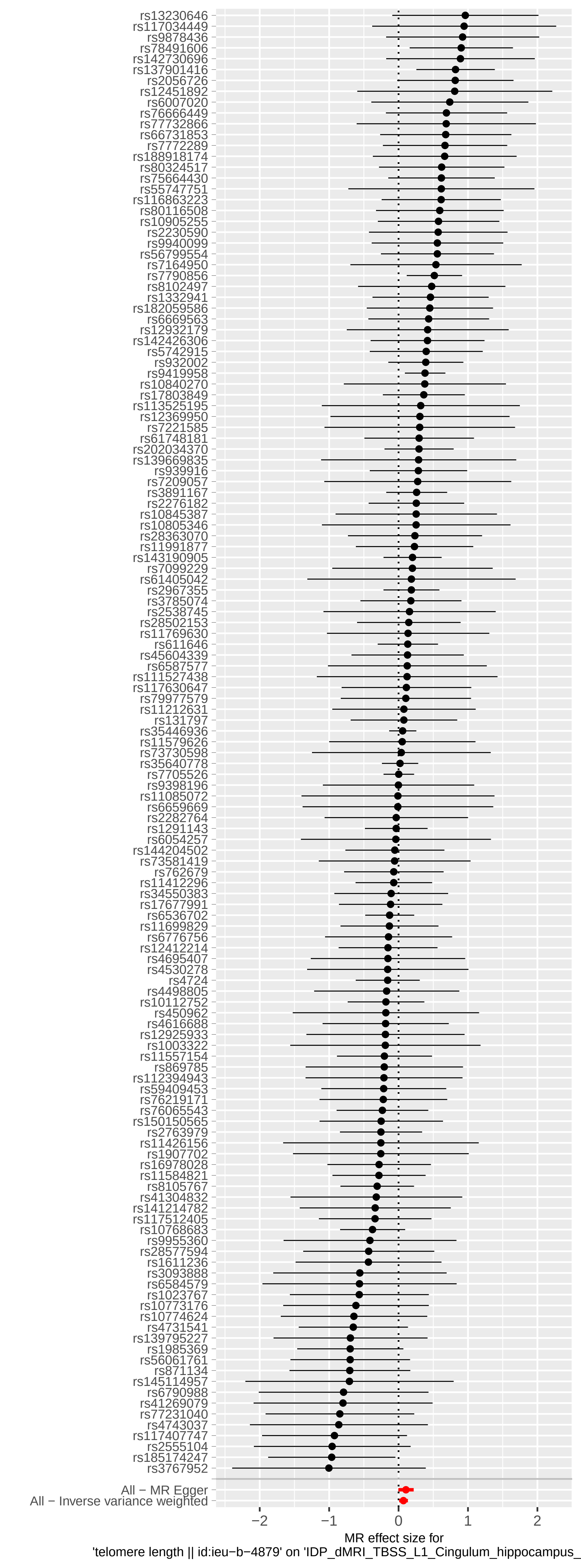
rs13230646
rs117034449
rs9878436
rs78491606
rs142730696
rs137901416
rs2056726
rs12451892
rs6007020
rs76666449
rs77732866
rs66731853
rs7772289
rs188918174
rs80324517
rs75664430
rs55747751
rs116863223
rs80116508
rs10905255
rs2230590
rs9940099
rs56799554
rs7164950
rs7790856
rs8102497
rs1332941
rs182059586
rs6669563
rs12932179
rs142426306
rs5742915
rs932002
rs9419958
rs10840270
rs17803849
rs113525195
rs12369950
rs7221585
rs61748181
rs202034370
rs139669835
rs939916
rs7209057
rs3891167
rs2276182
rs10845387
rs10805346
rs28363070
rs11991877
rs143190905
rs7099229
rs61405042
rs2967355
rs3785074
rs2538745
rs28502153
rs11769630
rs611646
rs45604339
rs6587577
rs111527438
rs117630647
rs79977579
rs11212631
rs131797
rs35446936
rs11579626
rs73730598
rs35640778
rs7705526
rs9398196
rs11085072
rs6659669
rs2282764
rs1291143
rs6054257
rs144204502
rs73581419
rs762679
rs11412296
rs34550383
rs17677991
rs6536702
rs11699829
rs6776756
rs12412214
rs4695407
rs4530278
rs4724
rs4498805
rs10112752
rs450962
rs4616688
rs12925933
rs1003322
rs11557154
rs869785
rs112394943
rs59409453
rs76219171
rs76065543
rs150150565
rs2763979
rs11426156
rs1907702
rs16978028
rs11584821
rs8105767
rs41304832
rs141214782
rs117512405
rs10768683
rs9955360
rs28577594
rs1611236
rs3093888
rs6584579
rs1023767
rs10773176
rs10774624
rs4731541
rs139795227
rs1985369
rs56061761
rs871134
rs145114957
rs6790988
rs41269079
rs77231040
rs4743037
rs117407747
rs2555104
rs185174247
rs3767952
All − MR Egger
All − Inverse variance weighted
−2
−1
0
1
2
MR effect size for
'telomere length || id:ieu−b−4879' on 'IDP_dMRI_TBSS_L1_Cingulum_hippocampus_
